# How do general practitioners experience providing care to refugees with mental health problems? A qualitative study from Denmark

**DOI:** 10.1186/1471-2296-14-17

**Published:** 2013-01-28

**Authors:** Natasja Koitzsch Jensen, Marie Norredam, Stefan Priebe, Allan Krasnik

**Affiliations:** 1 The Danish Research Centre for Migration, Ethnicity and Health, Section for Health Services Research, Department of Public Health, University of Copenhagen, Øster Farimagsgade 5, 1014, Copenhagen, Denmark; 2 Unit for Social and Community Psychiatry, Queen Mary University of London, Newham Centre for Mental Health, London, E13 8SP, United Kingdom

**Keywords:** Primary care, Refugees, Migration, Trans-cultural psychiatry, Mental health, Categorisation

## Abstract

**Background:**

Refugees are a particularly vulnerable group in relation to the development of mental illness and many may have been subjected to torture or other traumatic experiences. General practitioners are gatekeepers for access to several parts of the psychiatric system and knowledge of their patients’ refugee background is crucial to secure adequate care. The aim of this study is to investigate how general practitioners experience providing care to refugees with mental health problems.

**Methods:**

The study was conducted as part of an EU project on European Best Practices in Access, Quality and Appropriateness of Health Services for Immigrants in Europe (EUGATE). Semi-structured interviews were carried out with nine general practitioners in the vicinity of Copenhagen purposively selected from areas with a high proportion of immigrants. The analysis of the interviews is inspired by qualitative content analysis.

**Results:**

One of the main themes identified in the analysis is communication. This includes the use of professional interpreters and that communication entails more than sharing a common language. Quality of care is another theme that emerges and includes awareness of possible trauma history, limited possibilities for refugees to participate in certain treatments due to language barriers and feelings of hopelessness in the general practitioners. The general practitioners may also choose different referral pathways for refugees and they report that their patients lack understanding regarding the differences between psychological problems and physical symptoms.

**Conclusion:**

General practitioners experience that providing care to refugees differs from providing care for patients from the majority population. The different strategies employed by the general practitioners in the health care treatment of refugees may be the result of the great diversity in the organisation of general practice in Denmark and the lack of a national strategy in the health care management of refugees. The findings from this study suggest that the development of conversational models for general practitioners including points to be aware of in the treatment of refugee patients may serve as a support in the management of refugee patients in primary care.

## Background

The United Nations Convention Relating to the Status of Refugees became effective in Denmark as of 1954. Two years later, Denmark received its first refugees after joining the convention, namely, around 1,400 people from Hungary. In the 1950s and 1960s, refugees were primarily people who had escaped from the communist states in Eastern Europe, whereas in the 1970s, the refugees arriving in the country were mainly Asians driven out of Uganda, refugees from Chile and Vietnamese boat refugees [1]. Through the 1980s and 1990s, the refugee group was dominated by people who had escaped from conflicts in the Middle East, Sri Lanka, Somalia and the Balkans [2]. The number of positive asylum applications has fluctuated through the years, but the overall tendency has been a decline in the number of individuals granted protection in the last ten years [3,4]. In 2010, the largest groups of people seeking asylum in Denmark were from Afghanistan, Syria, Iran and Russia, and approximately 2,124 individuals obtained refugee or similar protection status [3]. In addition, Denmark receives 500 quota refugees per year as part of an agreement entered into with the United Nations High Commissioner for Refugees [1]. The societal discourse and the refugee politics of the Danish government have varied over the years [1,5]. In 1983, the Danish Aliens Act was changed and was regarded as one of the most humane and liberal alien acts in Europe [1,6]. It introduced measures such as granting refugees the right to asylum given that they fulfilled the conditions of the law, it extended the refugee concept to include de facto refugees, i.e., individuals who did not meet the requirements of the UN convention were still able to obtain asylum, an appeal authority was established for people who had had their asylum application rejected and refugees were able to claim family reunification. One year later, the world’s first rehabilitation centre for torture survivors was opened in Copenhagen. Though the principles in the asylum system are still the same, it has become more difficult to access Denmark [1,7]. For instance, the former government implemented changes in the Aliens Act in 2005 implying that the selection of quota refugees to a greater extent should be based on their integration potential assessed through parameters such as age, education, network and motivation [8] compared to an earlier focus based on who was most in need [1]. Denmark has a tax-financed health care system and the majority of services are offered free of charge [9]. Recognised refugees are enrolled with the National Register of Persons and, thereby, they are entitled to access health care services on the same terms as Danish citizens residing in the country and in the case they are traumatised, they are entitled to cost-free treatment in specialised rehabilitation centres [10].

Refugees are a particularly vulnerable group in relation to the development of mental illness [11]. Migration can be a stressful event regardless of the circumstances, but refugees are being forced to flee their countries and have often been exposed to events before, during and after migration that can influence their mental health [11-14]. Great variations in the prevalence of mental illness in refugees are reported in different studies, which is most likely due to heterogeneity in choice of study populations and measurement instruments [15]. However, in a meta-analysis of 20 studies, it was reported that approximately one in ten adult refugees settled in Western countries had post-traumatic stress disorder and it was concluded that they may be 10 times more likely to suffer from this disorder than an age-matched general American population. In addition, the results suggest that about one in 20 suffers from major depression and about one in 25 has generalised anxiety disorder. It is likely that many people have comorbid diagnoses in relation to these disorders [16]. In a recent study from Denmark, it was also concluded that refugees have an increased risk of mental illness in comparison with native-born Danes measured by the number of first time contacts with the psychiatric hospital system [17]. In spite of this, a qualitative study by Amnesty International, based on interviews with former asylum seekers having been subjected to torture, showed that this group underutilises psychiatric health care services though a great need exists. It is concluded in the study by Amnesty International that contact with health care services becomes less frequent after asylum seekers leave asylum centres and that referrals to relevant health care services are lacking. Even the former asylum seekers who are in contact with the regular health care system and would benefit from psychiatric treatment have never been in contact with the psychiatric health care system [18].

Recent studies in Denmark and Norway show that between 45–57.3% of the asylum seekers arriving in these countries have been subjected to torture [19,20]. However, even asylum seekers who have not experienced torture will often have experienced other traumatic events rendering them particularly vulnerable [19]. Survivors of torture may not easily share their stories on their own initiative [21]. Furthermore, several studies show that general practitioners are uncertain whether a refugee background is of importance in relation to the health of their patients [22-24] and that they are reluctant to initiate conversations about traumatic experiences in relation to the situation in their home countries, their escape and their life in exile [24].

In summary, refugees are a vulnerable group in relation to mental health problems and the seemingly scarce awareness of this subject on the side of the health professionals should be reason for concern. General practitioners are the gatekeepers for access to much psychiatric treatment, and knowledge of how they experience providing care to refugees is crucial in order to secure adequate treatment for this patient group. The aim of this study was to qualitatively explore issues identified by general practitioners as important in their experiences of providing care for refugees with mental health problems.

## Methods

The study was part of an EU project on Best Practices in Access, Quality and Appropriateness of Health Services for Immigrants in Europe (EUGATE). The study was carried out in 16 European countries and international findings from the study are presented elsewhere [25-27]. This study presents findings from a single country, namely Denmark, to be able to provide more in depth analysis in a specific country setting contrary to focusing on findings across European countries with diverging health care systems and experiences of immigration.

### Data generation

The data for the Danish part of the study were collected between September 2008 and January 2009. A total of 15 semi-structured interviews were conducted with general practitioners (9), emergency room physicians (3) and managers of psychiatric residential units (3). The participants were purposively selected from areas with high proportions of immigrants and chosen from three different hospital catchment areas representing urbanised areas in the vicinity of Copenhagen. Initially, the health professionals were informed about the study in a letter, and shortly after they were contacted by the researcher by phone in order to describe the study in further detail and inquire about their interest in participating in the study. The interview guide used in the interviews was developed by the EUGATE coordinating group, based with Queen Mary University in London, and was piloted in each of the participating countries. The interview guide was translated from English into Danish by the first author. The interviews took place at the workplace of the health professionals and were carried out by the first author. The health professionals were interviewed once and interviews lasted between 30 min and 1 ½ hour. Interviews were recorded on dictaphone and transcribed ad verbatim in Danish. Quotes selected to substantiate the analysis have later been translated into English for the purpose of this paper. The complete interview guide employed in the interviews can be found in the Additional file 1. This article builds on the interviews with general practitioners. The first part of the interviews focused on the experiences of general practitioners with delivery of care to immigrants in general, whereas the next part of the interview included vignettes with different immigration status. This article is based on a vignette describing a female refugee with poor Danish communication skills:

"A refugee woman, 39 years old, from Afghanistan, presents with headache, anxiety, sleeping problems and stomach ache. She has minimal command of the language of the host country. She brings her 12-year-old daughter along, who speaks the language of the host country very well."

The discussion points for the vignette focus on how treatment and further referral through the system differ from ethnic Danes and are presented in Table 1.

**Table 1:** Discussion points for the vignette

	
·	From your perspective, what are the differences, if any, in the treatment for this patient compared to a patient from the native-born population with a similar condition?
·	From the perspective of a patient, what do you think are the specific problems this patient would encounter that are different from those of a patient from the native-born population with a similar condition, and how would they be overcome?
·	What are the specific further pathways and treatment options, if any, for this patient that are different from those of a patient from the native-born population with a similar condition?
·	Would you use the daughter as an interpreter? If not, would you use the daughter as an interpreter if she was 18 years or older?

### Analysis

The analysis of the interviews is inspired by qualitative content analysis as described by Graneheim and Lundman [28]. This method was chosen as it is thoroughly described and allows for exploratory analysis of data compared to an analysis guided by a specific theory chosen a priori to the analysis. The approach is part of the hermeneutic tradition in which it is assumed that “reality” can be interpreted in various ways and that all understanding depends on subjective interpretation. Hence, even though the themes are presented separately we recognize they are interlinked and could have been presented differently. The interviews were read several times to obtain a sense of the whole. The text was then divided into meaning units, which were then condensed and assigned categories and themes in a process moving towards a higher level of abstraction. The creation of categories and themes took place as an iterative process with ongoing reflection and revision of categories and themes. The whole context of the interviews was considered concurrently throughout this process. The initial analysis was carried out by the first author, but presented to and discussed with co-authors and other researchers with a background in public health, medicine and anthropology as part of the analytic process. The presented themes reflect the concerns from the professionals’ point of view.

### Ethical permission and informed consent

According to Danish legislation ethical permission is only required for research making use of biological samples [29]. Hence, it is not requested for studies based on interviews such as the present one and ethical permission for this study has been waived by the Ethical Committee of the Capital Region of Denmark (file number H-3-2012-FSP40). The study was carried out in accordance with ethical principles of research in the health and social sciences [30,31] and in line with RATS guidelines for qualitative research [32]. Informed consent was obtained orally from all participants and they were ensured anonymity.

## Results

Two out of nine participants in the study were male and two of the general practitioners were immigrants themselves. They all owned or partially owned clinics in the vicinity of Copenhagen and from areas with high a percentage of immigrants in the population. The number of staff working in the clinics ranged from two to ten (Table 2). The themes presented in the analyses include communication, quality of care, referral pathways, and understandings of disease and expectations of treatment. A short overview of the main findings can be found in Table 3.

**Table 2:** Overview of participating general practitioners

**ID number**	**Sex**	**Origin**	**Immigrant**	**Organisation**	No. staff in the clinic*	**No. of immigrant staff**
**46**	Female	Denmark	No	Single practitioner	4	1
**47**	Female	Denmark	No	Single practitioner	3	0
**48**	Male	Denmark	No	Partnership practice	7	0
**49**	Female	Turkey	Yes	Single practitioner	3	2
**50**	Female	Denmark	No	Shared practice	5	3
**51**	Female	Denmark	No	Single practitioner	2	0
**52**	Female	Denmark	No	Shared practice	10	0
**53**	Male	Afghanistan	Yes	Single practitioner	4	3
**54**	Female	Denmark	No	Shared practice	4	0

*The number includes doctors, nurses and administrative staff. Not all staff work full time in the clinic.

**Table 3:** Main findings

**Theme**	
**Communication**	
·	The general practitioners prefer to use professional interpreters
·	Communication entails more than sharing a common language
**Quality of care**	
·	The general practitioners show limited awareness of past trauma among refugee patients
·	The general practitioners may feel reluctant to initiate certain types of treatment due to language barriers in refugee patients
·	The general practitioners may feel powerless in the treatment of refugee patients
**Referral pathways**	
·	The general practitioners may refer refugee patients to specialised treatment centres for traumatised refugees
**Expectations and understanding of disease**	
·	The refugee patients may lack an understanding of the connection between psychological problems and physical symptoms
·	The refugee patients may exhibit different expectations of treatment

### Communication

The general practitioners all expressed that they prefer to use professional interpreters instead of using family members in the consultation. One of the general practitioners explained why a professional interpreter is preferred: *“I have sometimes experienced that a patient has insisted on her/his own interpreter. Sometimes it is okay, but in the majority of the cases it is better with the authorised interpreters since they are more familiar with the medical terminology. So it is always a poorer consultation. It is typically the family being used and I feel they shouldn’t be there at all” (ID 50, female GP).* In spite of this, a few of the general practitioners expressed that they, every now and then, make use of family interpreters when the patient and the relative both insist. The general practitioners explained that they only use children for interpretation in very trivial matters such as booking a new appointment or explaining that blood pressure is being measured. Furthermore, they explained that a lack of interpreter will often result in arranging a new consultation, which can lead to considerable frustration for the patient and the risk of misunderstandings as the patient may feel rejected in his/her initial contact with the health care services. One of the general practitioners noted: “*Being able to talk together is not always dependent on the words, but has more to do with whether you are on the same wavelength as somebody. So that particular patient, I don’t know if it is the pure language abilities. It may depend more on a feeling of whether she thinks I am an idiot or she feels that I am inpatient. In that way it is not so different from Danish patients (…). To feel understood is not always a technical language thing - you can talk the same language and still not be able to understand one another” (ID 51, female GP).* This general practitioner makes the point that communication between the professional and the patient entails more than sharing a common language.

### Quality of care

Three of the general practitioners explained that it is important to pay attention to whether the patient may have been traumatised. One of them said: *“Then I just mentioned the thing with her having some traumas such as rape or having experienced a war situation; I hardly think about that when it is an ethnic Dane. There is a different background to pay attention to” (ID 48, male GP).* Another general practitioner – who has an immigrant background himself – also stressed the importance of paying attention to the potential trauma of refugee patients, but still focus more on the commonalities between a female refugee and an ethnic Danish woman. He stated that it is important to be aware that ethnic Danish women may also have been traumatised. A few of the participants explained that participation in certain types of treatments may be restricted due to the language barrier. One of the general practitioners explained how language may hinder access to treatment: *“She may be entitled to a psychologist or other services through the health insurance, but I am thinking ‘how do you find a psychologist that speaks Afghani?’. If you need the conversation as a tool, it requires that the professional knows the language. Otherwise it doesn’t make any sense (…). There are some options which are not possible for this type of patient. I cannot just give her a referral for a psychologist and then say go home and book an appointment online as I would with a Danish patient. The options are there, but you cannot make use of them” (ID 51, female GP).* This exemplifies how general practitioners may not be able to make use of certain external treatment facilities in the health care system for this group of patients. In addition, it was also expressed that working with conversational therapy in the general practice is also limited for patients that do not speak the language of the host country unless they are able to work with extremely skilled interpreters. The general practitioners may still listen to the story of the patient, but expressed they will not be able to offer therapy in the same way as with a patient that spoke the Danish language. Treating refugees could also give rise to feelings of powerlessness in the general practitioners. One of the general practitioners expressed her view of immigrant patients with psychosomatic diseases in the following way: *“In reality there are no prospects at all. It is a question of finding a way to live as well as possible. It is a shame because there is – or has been – so much potential in these people, which has not been made use of” (ID 50, female GP)*. The feelings of how powerless the general practitioners may feel were clearly conveyed in this quote. This may again impact the treatment possibilities the general practitioner envision for the refugee patients.

### Referral pathways

The general practitioners explained how referral pathways may differ between refugee patients and ethnic Danish patients. Several of the general practitioners explained that they would refer the refugee patient to a specialised service for traumatised refugees, as the refugee patients may be too complicated for them to handle on their own. One of them said: “*(…) the question is whether she has PTSD. If she did I would start her treatment and then refer her to the Centre for Traumatised Refugees at Gentofte Hospital. They do a full assessment of the patient and the treatment in a relatively short time (ID 49, female GP).* The general practitioners may choose to refer the refugees with mental health problems to more specialised treatment facilities. Patients with mental health problems are described by the general practitioners as very resource demanding regardless of being a refugee or an ethnic Dane. One of the general practitioners expressed that she may be better equipped to handle a traumatised patient who is an ethnic Dane, whereas another, again, stressed commonalities between patients with psychiatric problems: *“I would still say that there is no difference for me here, because I think it depends on the status of the patient. If it is an ethnic Dane that had been traumatised, it would also require a lot of time and effort, such as conversations with a psychologist and all sorts of things, and that holds true for the other patient as well. I need to have more conversations as well and I need lots of time. But again, with the patients suffering from psychological problems, they are lost for life. You cannot refer them much further, you cannot do a lot for them” (ID 53, male GP).* The commonalities between refugee and ethnic Danish patients were stressed by this general practitioner, but the quote also expresses that the general practitioner does not have much hope for any improvement in the situation even if referring the patients for further treatment.

### Understandings of disease and expectations for treatment

Several of the general practitioners expressed that the patient and the professional may not have the same understanding of health and disease. Some of the professionals expressed that refugee patients may have difficulties understanding that psychological and social problems may also lead to the presentation of physical symptoms. One of the general practitioners said: “*She probably has a hard time putting her experience of physical problems into a context. She may be scared that if she refers to it as something related to her social life instead of physical illness she may feel it is all wrong. That is probably different compared to ethnic Danes” (ID 46, female GP).* In addition, the refugee patients may also carry different and very high expectations for the professionals. The general practitioners expressed that such expectations may lead to demands for being cured really quickly and a reliance on treatment involving medication. One of the general practitioners expressed how the patients’ expectations of receiving medicine may clash with her understanding of the needed treatment: *“… and when people show up with their ‘hurting in life’ symptoms because they have a hard time adjusting to the country or because they have some war trauma which hasn’t been processed properly or they miss their family back home and then I say I don’t have a pill to help with this. I have examined you and I don’t think you have a severe depression, but in your life situation everyone would have a really hard time. […]. You can come and tell your story here, even if you can’t tell it anywhere else, but you don’t need medication” (ID 48, male GP).* The general practitioners explained how they may be met with patients presenting problems outside their area of expertise and that they are not always able to live up to the expectations uttered by patients. One of them said: *“Sometimes it may be unhappy marriages or problems with the children which are outside my area of action. As I sometimes say, ‘I am only a doctor’. Sometimes there are far greater expectations than what you can honour” (ID 50, female GP).* The quote underlines that the general practitioners are not only presented with medical problems, but problems in the social sphere in the lives of their patients. The general practitioners find it difficult to manage such general life issues presented by their patients.

## Discussion

### Summary of main findings

The current study investigated general practitioners’ experiences with providing care to refugees with mental health problems. Thereby, we gained insight into areas that may pose problems in the delivery of care for this particular group. One of the main themes in the delivery of care to refugees was communication. This theme included considerations related to the use of interpreters, but also that communication with patients entails more than simply speaking the same language. Another theme in the analysis was quality of care, which included that health professionals should be aware of a possible history of trauma among refugee patients, limited possibilities for treatments such as conversational therapy and psychologist due to language problems and a feeling of powerlessness in the general practitioners as they do not have much hope for improvement of refugee patients with mental health problems. Different referral possibilities for refugee patients were also mentioned by the general practitioners as important in the delivery of care. The health professionals also expressed that the refugee patients may have more difficulties understanding that psychological problems can lead to physical symptoms. The general practitioners also experienced that the patients have an expectation of receiving medication for problems related to their social circumstances and great expectations of the professional in terms of solving social problems.

### Refugees and access to health care treatment

Having a background as a refugee is associated with a higher risk for developing a mental illness [14-16]. However, it has previously been found that general practitioners are unaware of the implications of patients having a refugee background and that they often refrain from enquiring further about the refugee situation [24]. In addition, there is also evidence that indicates that torture and other traumatic incidents experienced by the patients may be missed by the general practitioners [22,23]. Contrary to these findings, some of the health professionals in our study expressed that they are aware of the importance of considering a traumatic background in regards to their refugee patients. However, only a few of the general practitioners who were interviewed expressed this awareness. The general practitioners also expressed feelings of resignation in relation to the effects of treatment of refugees with mental health problems. They expressed that they would rather refer the patient to specialised treatment in rehabilitation centres for traumatised refugees instead of managing the treatment by themselves. Additional treatment possibilities are available for refugees that have been traumatised, whereas there may be a reluctance to initiate conversational therapy in the primary care setting or refer refugees for treatment options such as psychologists due to language problems.

### Differentiating between psychological and physical symptoms

The general practitioners mentioned that the refugee patients have difficulty understanding the connection between psychological problems and physical symptoms. Naturally, in our study this is the general practitioners’ assessment of the patients. However, in a study in Great Britain it was reported that patients with a Punjabi background were less likely to acknowledge an emotional origin to the physical symptoms they experienced [33], which supports the experience of the general practitioners in our study. Interestingly, the general practitioners in our study do not reflect on their own ability to distinguish between psychological and physical problems. Other literature in the area of migration and mental health has shown that health professionals may experience problems with diagnosing patients from a different cultural background. When the general practitioners in the aforementioned study were to assess British and Punjabi patients, the help-seeking patients of British origin were more likely to be diagnosed correctly. The Punjabi patients suffering from a common mental disorder were more likely to be assessed as having “physical and somatic symptoms” or “sub-clinical disorders” [33]. Literature from mental health units has also highlighted difficulties with diagnosing mental health problems in patients from different cultural backgrounds [34]. This may be due to language problems, different belief systems making it difficult to differentiate between certain beliefs and symptoms of a mental illness or trying to distinguish post traumatic stress disorder from psychotic symptoms in immigrant patients [35]. Problems with correct diagnosing of patients in the area of mental health have also been reported with other psychiatric diseases such as dementia [36-38] and schizophrenia [39].

### Variations in strategies towards refugees

Our findings reflect that the general practitioners employ many different strategies in the management of refugee patients. This may be in relation to the use of interpreters, their approach to refugee patients and use of different referral pathways in the health care system. These varying strategies may be a result of the organisation of the health care services and more specifically the general practitioners. In Denmark, general practitioners run private practices though they receive the main part of their income from public funds from the health insurance managed by the regional authorities [9,40]. They have independent practices and there is no national strategy for general practitioners to follow regarding health care management of refugees. In a recent study, it was found that there were large variations in the health care reception of quota refugees in municipalities in Denmark. Though the organisational structures of the municipalities are different from general practitioners, this lack of a national strategy led to very different practices between individual municipalities [41]. The National Board of Health has minimal recommendations regarding the health management of refugees, which mainly focus on the use of professional interpreters. The lack of a national strategy in combination with the independent organisation of general practitioners may account for the different approaches seen amongst the general practitioners in our study, as well as some of the frustration and hopelessness some of the general practitioners express. Health professionals in other medical fields have also been found to experience uncertainty in their work with patients with an ethnicity different from their own. The experienced uncertainty was disempowering for the health professionals and led to inertia in their practice despite trying to do the best for their patients [42].

### Categorisation

Being a refugee can have implications in relation to the development of mental illness [16] and it is important that health professionals are aware of possible traumas [43]. In this study we examined in what situations general practitioners find differences in the delivery of care for refugees and ethnic Danes. The division of people into categories takes place every day and ascribed categories can give rise to both different entitlements and limitations, e.g., limited health care access for undocumented immigrants and extended access for certain services for traumatised refugees. This being said, it is important to remember that categories are socially created. Though the world is essentially continuous, it is experienced in discrete chunks, which can be described in terms of lumping and splitting [44]. With lumping, similar things are grouped together and their perceived similarities are allowed to outweigh differences within the group, and we envision the people in the group as relatively homogeneous. Splitting on the other hand involves widening the perceived differences between the created categories [44]. In the interviews, we asked were there differences in the provision of care between refugees and ethnic Danish patients, although some participants noted that sometimes the difference is not so large after all. One of the general practitioners, who is an immigrant himself, focused particularly on the commonalities between patients and was critical towards the use of the category of refugees in the study. Categories are not given by nature, but are socially created [44,45] and they are produced and reproduced in human interactions [46]. Using a range of cues, verbal and non-verbal, identification combines criteria of similarity and difference in order to locate self and others on a ‘social map’ of relationships and collectivities [45]. The categorisation of patients takes place in an institutional context where power structures such as layperson-specialist, minority-majority, and sick-well are at stake [47]. In the meeting between a patient and health professional, it is the professional who is able to provide access to further services in the health care system. Categorising people entails the risk of focusing on the differences between the groups instead of the commonalities and also ignoring differences existing within the assigned categories [44]. In this study design, it is assumed that being a refugee implies that people in this category have more in common than for instance people with the same educational level. Making the category of refugee the main focus of attention implies that the category can encompass very well educated refugees from a large and modern city to refugees who have spent their entire lives in a refugee camp or in a small village without any schooling; this assumption was also challenged by one of the participants in the study.

### Methodological reflections

The health professionals selected to participate in the study were all from areas with high proportions of immigrants in the vicinity of Copenhagen. Therefore, it is expected that all of the general practitioners had vast experience with immigrant and refugee patients, which is considered a strength of the study as it ensures knowledge of the target group. However, it could also be viewed as a limitation as the results might have been different had we interviewed general practitioners from areas with very little exposure to immigrant and refugee patients. Another limitation includes that the analysis in this study is based on a vignette of a refugee patient. It is a situation that describes an isolated consultation between a refugee patient and a general practitioner which may influence the generalisability of the findings as the general practitioners will often have had a continuous relationship with many of their patients, and in a regular setting will be able to estimate body language and expressions of the patients. Furthermore, it is not possible to know who the professionals were thinking about when participating in the interview. It may have been refugees in general or there may have been a particular patient that has greatly affected the general practitioner such that the practitioner had this person in mind during the interview. The interview started out with focusing on immigrants in general and this may have influenced their way of thinking when engaging in the vignettes focusing on more specific groups of immigrants. During the interviews there was a focus on creating an atmosphere in which the participant could express his/her opinions freely and without any judgments from the interviewer. However, immigration and psychiatry are both fields which receive much attention from politicians and are frequently debated in the media and, therefore, there is a risk of social desirability in the answers of the general practitioners. This interview study provides insight into how the general practitioners experience differences in providing care to refugee patients compared to ethnic Danish patients. Investigating the area from a quantitative angle could provide insight into how many general practitioners are aware of the importance of a refugee background in relation to the health status of a patient. In addition, the patient perspective would also be an important approach to investigate. The patient perspective can easily be disregarded in research focusing on mental health care, but it is still highly relevant to investigate how patients experience the care they receive.

## Conclusion

The general practitioners experience that providing care for refugees differs from providing care for the majority population in several ways. Our findings reflect that the general practitioners employ very different strategies in the care of refugee patients, which may be a result of a great diversity in how general practice in Denmark is organised and the lack of a national strategy in the general health care management of refugees. The findings from this study suggest that there is an increased need for general practitioners to be aware of potential traumas experienced by refugee patients, but also leave room for taking individual differences into account in the consultation. This could be attained by the development of conversational models for general practitioners including points to be aware of in the treatment of refugee patients. This may serve as a support in the health care management of refugee patients, but at the same time does not disregard the resources of individual refugee patients.

## Competing interests

The authors declare that they have no competing interests.

## Authors’ contributions

NKJ has made substantial contributions to the design of the study and has carried out the data collection, the data analysis, interpretation of data, and drafted the manuscript. MN has contributed to the analysis and has revised the manuscript critically for important intellectual content. SP and AK have made substantial contributions to the design and have revised the manuscript critically for important intellectual content. All authors have read and approved the final manuscript.

## Pre-publication history

The pre-publication history for this paper can be accessed here:

http://www.biomedcentral.com/1471-2296/14/17/prepub

## Supplementary Material

Additional file 1 European Best Practices in Access, Quality and Appropriateness of Health Services for Immigrants in Europe. Click here for file
