# Supplementary material for: How do general practitioners experience providing care to refugees with mental health problems? A qualitative study from Denmark
Source: BMC Fam Pract. 2013 Jan 28;14:17. doi: 10.1186/1471-2296-14-17 (PMC3568406; doi:10.1186/1471-2296-14-17)
Supplement: Additional file 1 — European Best Practices in Access, Quality and Appropriateness of Health Services for Immigrants in Europe. [file 1471-2296-14-17-S1.pdf]

**European Best Practices in Access, Quality and Appropriateness of Health Services for Immigrants in Europe**

# EUGATE QUESTIONNAIRE ON SERVICES

Dear Practitioner,

Following on from our recent communication, we are sending a brief overview of the EUGATE study.

***What is the purpose of this study?*** Meeting the health needs of the increasing groups of immigrants is a major challenge to public health throughout Europe. EUGATE aims to consolidate the currently fragmented knowledge in the field and identify the best practice of health care for different immigrant populations across 16 EU countries. The ultimate goal of EUGATE is to define guidelines for best practice.

***What will my participation involve?*** As part of the study, we conduct face-to-face interviews with practitioners in health services in each country. Your service has been selected for the study as it operates in an area with a high proportion of immigrants. The manager or a head of your department has already agreed for this survey to take place. The interview will cover different aspects of care for immigrants. The questionnaire includes information which may be readily available. However, if you do not have access to the requested information, provision of estimates will suffice. We envisage that the questionnaire will take no longer than 40 minutes. If, however, you do not have access to such information, provision of estimates will suffice.

***Will my taking part in this study be kept confidential?*** If you agree to take part, all information you provide will be confidential and all data will be anonymised. Your name or the name of the service will not be recorded in our database.

***What is in it for me?*** There is no direct benefit for you, but we would be most grateful if you would support this research. If you are interested, we will be happy to send you a report with the results of the survey after its completion in 2010.

If you have any questions concerning EUGATE or this questionnaire, please do not hesitate to contact [Mr X], scientific coordinator EUGATE Project, [contact details].
[*to be completed by each centre*!]

Thank you for your time.

EUGATE team

(For researcher use only: ID Number ___________________)

# PART 1 – GENERAL INFORMATION

***To be filled in by the researcher***

## 1. Concerning the service

| | | | | | | |
|---|---|---|---|---|---|---|
| 1.1. Identified area | | | | | | |
| 1.2. Type of service | | | | | | |
| 1.3. Professional role of main person interviewed using this questionnaire: | | | | | | |
| Clinician/ Practitioner | | Nurse | | Administration/ Manager | | Other (please specify) |
| 1.4 Roles of other people the research may have contacted to complete the questionnaire (indicate the number of clinicians, nurses, administration or other personnel contacted) | | | | | | |
| Clinician/ Practitioner | | Nurse | | Administration/ Manager | | Other (please specify) |

## 2. Patient characteristics

**Within this section of the questionnaire, immigrants are defined as any person born outside *[insert the name of your country]* (excluding persons who were born abroad but are *[insert country's nationality]* nationals by birth. They can be EU citizens or from other parts of the world.**

**Below you will find definitions for individual target immigrant groups. If data or estimates are also available specifically for immigrants and the target immigrant groups aged 18-65, please also record this data.**

**Other useful definitions:**

**Regular immigrants** are those born outside *[insert the name of your country]* (excluding persons who were born abroad but are *[insert country's nationality]* nationals by birth and who are legally residing in the territory of a Member State, e.g. migrant workers, long-term residents (not tourists).

**Asylum seekers** are persons who have left their country of origin, have applied for asylum in another country, and are awaiting a decision on their application. They hope to obtain refugee status or protection on other humanitarian grounds in order to benefit from the legal protection and material assistance which is an automatic part of such protection.

A **refugee** is defined in Article 1 of the 1951 Convention as a person who "owing to a well-founded fear of being persecuted for reasons of race, religion, nationality, membership of a particular social group, or political opinion, is outside the country of his nationality, and is unable to or, owing to such fear, is unwilling to avail himself of the protection of that country… In EU Member States, recognized refugees are entitled to full access to national health systems on the same basis as nationals, mostly by virtue of the fact that they are granted permanent residence status and the rights that accompany this, or are granted the same rights as nationals under the asylum law of the country in question.

**Victims of human being trafficking** are persons trafficked for sexual exploitation, for labour, for begging and delinquency, for adoption or for any other form of exploitation from one country to another.

**Illegal or irregular immigrants** are those persons who have not been granted permission to enter or to stay in a given country.

**2.1. Does your service have a database containing patient records?**

☐ Yes ☐ No

| **2.2. Number of patients using the service** | Unknown | Value based on (please fill in only one field) | |
|---|---|---|---|
| | | Number based on data | Estimated number |
| 2.2.1. How many patients have used this service in the last 12 months (or any other 12 months for which data is available)?<br><br>Please specify to what data the number refers (e.g. caseloads/registers, treatment episodes, consultations, admissions):<br><br>Specify _______________ | | | |

| **2.3. Number of immigrant patients using the service** | | Unknown | Value based on (please fill in only one field for each question) | | | |
|---|---|---|---|---|---|---|
| | | | Number based on data | | Estimated number | |
| | | | Number OR % | | Number OR % | |
| 2.3.1. How many of these are immigrant patients? | | | | | | |
| 2.3.2. If known, how many patients from the following immigrant groups used this service in the last 12 months?<br>If entering %, please calculate % of the total number of patients. | | | | | | |
| | Regular immigrants | | | | | |
| | Asylum seekers | | | | | |
| | Refugees | | | | | |
| | Victims of human trafficking | | | | | |
| | Illegal immigrants | | | | | |

**2.4. What are the most frequent 5 nationalities of your immigrant patients? Please record the groups in order of their frequency (with '1' being the most frequent).**

1. ______________________________
2. ______________________________
3. ______________________________
4. ______________________________
5. ______________________________

☐ **Classification based on records or computerized data** ☐ **Interviewee approximate estimate**

## 3. Existence of specific departments, programmes and staff for immigrant patients

**3.1. Does your service have any specific departments or part of the service dedicated to any of the following immigrant groups? (Please tick all that apply)**

☐ Yes ☐ No

**If no, skip to 3.2.**

**If yes, please specify type of department for relevant immigrant group(s) :**

| | |
|---|---|
| All immigrants (not differentiating between immigrant groups) | |
| Regular immigrants | |
| Asylum seekers | |
| Refugees | |
| Victims of human being trafficking | |
| Illegal immigrants | |

**3.2. Does your service offer any specific programmes for any of the following groups that are different from those offered to other service users?**

☐ Yes ☐ No

**If no, skip to 3.3.**

| | **If yes, please specify type of the programme for relevant immigrant group(s):** |
|---|---|
| All immigrants (not differentiating between immigrant groups) | |
| Regular immigrants | |
| Asylum seekers | |
| Refugees | |
| Victims of human being trafficking | |
| Illegal immigrants | |

**3.3. Does your service have any specific written policies or guidelines for any of the following groups that are different from those applied to other service users?**

☐ Yes ☐ No

**If no, skip to 3.4.**

| | **If yes, please specify main points of the policy or guideline for relevant immigrant group (s) :** |
|---|---|
| All immigrants (not differentiating between immigrant groups) | |
| Regular immigrants | |
| Asylum seekers | |
| Refugees | |
| Victims of human being trafficking | |
| Illegal immigrants | |

**3.4. Does your service have any specific staff dedicated to any of the following groups? (Please tick all that apply)**

☐ Yes ☐ No

**If no, skip to 3.5.**

| | **If yes, please specify staff role and main tasks for relevant immigrant group(s):** |
|---|---|
| All immigrants (not differentiating between immigrant groups) | |
| Regular immigrants | |
| Asylum seekers | |
| Refugees | |
| Victims of human being trafficking | |
| Illegal immigrants | |

**3.5. How many staff are employed within your service?**

**3.5.1. Does your service employ any immigrants (born outside the country) among your health staff?**

☐ Yes ☐ No

**If no, skip to question 3.6.**

**3.5.1.1. If yes, approximately how many?**

**3.5.1.2. What are the 3 most frequent nationalities (backgrounds) among immigrant employed at your service?**

**1.**
**2.**
**3.**

☐ **Classification based on records or computerized data** ☐ **Interviewee approximate estimate**

**3.6. Does your service provide interpreting services for immigrant patients when necessary?**

☐ Always ☐ Sometimes ☐ Never

**If 'never', skip to 4.1.**

**3.6.1. If yes, what type of interpreting service does your service provide? Tick all that apply.**

☐ Direct ☐ Telephone ☐ Other (specify) ___________________

**3.6.2. On average, how long does it take to arrange an interpreter?**

| | | | |
|---|---|---|---|
| Direct: | Days________ | Hours________ | Minutes_______ |
| Telephone | Days________ | Hours________ | Minutes_______ |
| Other | Days________ | Hours________ | Minutes_______ |

## 4. Evaluation

**4.1. Does your service have any evaluation and quality management systems in general (focus groups, feedback questionnaires, etc)?**

☐ **Yes**

☐ **No** **If not, please skip to question 4.3.**

**4.1.1. If yes, please specify the method(s) used (please tick all that apply):**

| **Method** | **Specifically for immigrant health care** | | **Not specifically for immigrant health care** | |
|---|---|---|---|---|
| | Yes | No | Yes | No |
| Anonymous satisfaction questionnaire | | | | |
| Suggestion box | | | | |
| Focus groups | | | | |
| Auditing by independent auditors | | | | |
| User representatives on consultative committees | | | | |
| User representatives on decision-making committees | | | | |
| Other | | | | |
| Please specify other | | | | |

**4.2. Are the results of these evaluations publicly available i.e. accessible to anyone wishing to access them?**

☐ Yes ☐ No

**4.3. Are immigrant patients involved in the management and/ or delivery of the service?**

| | General administration/ management (please tick) | | Delivery/ direct contact with clients (please tick) | |
|---|---|---|---|---|
| | Yes | No | Yes | No |
| Paid role | | | | |
| Unpaid role | | | | |

## PART 2 - GENERAL EXPERIENCES OF THE PERSON INTERVIEWED

*To be filled in by the researcher*

**This section consists of 5 open questions relating to the general experiences of practitioners when caring for immigrant patients. When answering these questions, please focus on immigrants who are a) born outside the country of current residence, b) aged 18-65 years, and c) arrived in the country within the last 5 years.**

**Do you accept that your answers are audio-recorded?**

☐ **Yes** ☐ **No**

| |
|---|
| **1. From your perspective, what are the specific problems for you in the care of immigrant patient in your service that you would not have in the care of a patient with a similar condition from the indigenous population?** |
| **2. From the perspective of a patient, what do you think are the specific problems faced by an immigrant patient coming into your service that are different from those faced by a patient with a similar condition coming from the indigenous population (e.g. communication)?** |

| |
|---|
| **3.** **In your experience, what are the strengths of your service in the care of immigrants?** |
| **4.** **What would improve the care for immigrants in your service?** |
| **5.** **EUGATE aims to create a repertoire of best practice models for immigrant care in Europe. Please indicate three services in your field of work (other than your service) that you would recommend as models of best practice in care provision for immigrants in your city. For each of these services, please give one or two reasons why you recommend them.** |

## PART 3 – CASE VIGNETTES

*To be filled in by the researcher*

**PRIMARY CARE**

| *Vignette 1 – illegal immigrant* |
|---|
| A male, 28 years old, coming from *[insert country]*, presents with pain when urinating and has a slight fever. He does not speak any language that the doctor understands. He has no insurance, no identification and no residency permit. |
| **1. From your perspective, what are the differences, if any, in the treatment for this patient compared to a patient with a similar condition from the indigenous population?** |
| **2. From the perspective of a patient, what do you think are the specific problems this patient would encounter that are different from those of a patient with a similar condition from the indigenous population, and how would they be overcome?** |
| **3. What are the specific further pathways and treatment options, if any, for this patient that are different from those of a patient with a similar condition from the indigenous population?** |
| **4. Would you inform the police and/or other authorities?** |

| *Vignette 2 – refugee* |
|---|
| A refugee woman, 39 years old, from *[insert country]*, presents with headache, anxiety, sleeping problems and stomach ache. She has very little command of the language of the host country. She brings her 12 years old daughter along, who speaks the language of the host country very well. |
| **1. From your perspective, what are the differences, if any, in the treatment for this patient compared to a patient with a similar condition from the indigenous population?** |
| **2. From the perspective of a patient, what do you think are the specific problems this patient would encounter that are different from those of a patient with a similar condition from the indigenous population, and how would they be overcome?** |
| **3. What are the specific further pathways and treatment options, if any, for this patient that are different from those of a patient with a similar condition from the indigenous population?** |

4. **Would you use the daughter as an interpreter?**

    a. **If no, would you use the daughter as an interpreter if she was 18 years old or over?**

| *Vignette 3 – labour immigrant* |
|---|
| A *[insert nationality]* woman, 40 years old, labour immigrant, widow, with two children 10 and 15 years old, asks for medication for her lower back pain. She speaks the language of the host country reasonably well, is working legally in a cleaning company and wants to go back to work urgently. |
| **1. From your perspective, what are the differences, if any, in the treatment for this patient compared to a patient with a similar condition from the indigenous population?** |
| **2. From the perspective of a patient, what do you think are the specific problems this patient would encounter that are different from those of a patient with a similar condition from the indigenous population, and how would they be overcome?** |
| **3. What are the specific further pathways and treatment options, if any, for this patient that are different from those of a patient with a similar condition from the indigenous population?** |

# European Best Practices in Access, Quality and Appropriateness of Health Services for Immigrants in Europe

**PART 3 – CASE VIGNETTES**

**ACCIDENT AND EMERGENCY DEPARTMENTS**

| ***Vignette 1 – illegal immigrant*** |
|---|
| The patient arrived in the host country as an illegal immigrant about 1 year ago. He is 25 yrs of age and of *[insert country]* origin. He does not speak any language that the A&E staff understand and presents with an intense lower abdominal pain. |
| **1. From your perspective, what are the differences, if any, in the treatment for this patient compared to a patient with a similar condition from the indigenous population?** |
| **2. From the perspective of a patient, what do you think are the specific problems this patient would encounter that are different from those of a patient with a similar condition from the indigenous population, and how would they be overcome?** |
| **3. What are the specific further pathways and treatment options, if any, for this patient that are different from those of a patient with a similar condition from the indigenous population?** |
| **4. Would you inform the police and/or other authorities?** |

| ***Vignette 2 – refugee*** |
|---|
| The female patient is 19 yrs of age and arrived from *[insert a country]* 10 months ago. She has refugee status and speaks only *[insert mother tongue]* and a few words of English. She is in her fifth month of pregnancy and has a serious complication (pre-eclampsia). She is reluctant to be examined by a male doctor. |
| **1. From your perspective, what are the differences, if any, in the treatment for this patient compared to a patient with a similar condition from the indigenous population?** |
| **2. From the perspective of a patient, what do you think are the specific problems this patient would encounter that are different from those of a patient with a similar condition from the indigenous population, and how would they be overcome?** |
| **3. What are the specific further pathways and treatment options, if any, for this patient that are different from those of a patient with a similar condition from the indigenous population?** |
| **4. Would it be arranged for a female doctor to examine her?** |

| |
|---|
| |

| *Vignette 3 – labour immigrant* |
|---|
| The male patient is 35 yrs of age and arrived from *[insert country]* two years ago. He has a regular residence permit. He was brought to A&E by the police because of his aggressive behaviour following heavy drinking. He suffered external head injuries in a fight. He is fully conscious and accessible for examination. |
| **1. From your perspective, what are the differences, if any, in the treatment for this patient compared to a patient with a similar condition from the indigenous population?** |
| **2. From the perspective of a patient, what do you think are the specific problems this patient would encounter that are different from those of a patient with a similar condition from the indigenous population, and how would they be overcome?** |
| **3. What are the specific further pathways and treatment options, if any, for this patient that are different from those of a patient with a similar condition from the indigenous population?** |

# European Best Practices in Access, Quality and Appropriateness of Health Services for Immigrants in Europe

**PART 3 – CASE VIGNETTES**

**MENTAL HEALTH SERVICES**

| ***Vignette 1 – illegal immigrant*** |
|---|
| The patient arrived in the host country as an illegal immigrant about 1 year ago. She is 25 yrs of age and of *[insert nationality]* origin. She does not speak the language of the host country, has no social contacts and appears severely depressed. |
| **1. From your perspective, what are the differences, if any, in the treatment for this patient compared to a patient with a similar condition from the indigenous population?** |
| **2. From the perspective of a patient, what do you think are the specific problems this patient would encounter that are different from those of a patient with a similar condition from the indigenous population, and how would they be overcome?** |
| **3. What are the specific further pathways and treatment options, if any, for this patient that are different from those of a patient with a similar condition from the indigenous population?** |
| **4. Is this scenario at all possible, or are there barriers preventing illegal immigrants from using your service?** |

| ***Vignette 2 – refugee*** |
|---|
| The male patient is 22 years of age, came to the host country from *[insert country]* a year ago and has refugee status. He speaks a few words of the language of the host country. He appears to have persistent auditory hallucinations and feel persecuted. |
| **1. From your perspective, what are the differences, if any, in the treatment for this patient compared to a patient with a similar condition from the indigenous population?** |
| **2. From the perspective of a patient, what do you think are the specific problems this patient would encounter that are different from those of a patient with a similar condition from the indigenous population, and how would they be overcome?** |
| **3. What are the specific further pathways and treatment options, if any, for this patient that are different from those of a patient with a similar condition from the indigenous population?** |
| **4. Would you encourage the patient to join an organisation for refugees from the same country?** |

| *Vignette 3 – labour immigrant* |
| --- |
| The female patient is 45 yrs of age and arrived from *[insert country]* two years ago. She has a regular residence permit, speaks the language of the host country well and suffers from a bipolar disorder with frequent and prolonged manic episodes. |
| **1. From your perspective, what are the differences, if any, in the treatment for this patient compared to a patient with a similar condition from the indigenous population?** |
| **2. From the perspective of a patient, what do you think are the specific problems this patient would encounter that are different from those of a patient with a similar condition from the indigenous population, and how would they be overcome?** |
| **3. What are the specific further pathways and treatment options, if any, for this patient that are different from those of a patient with a similar condition from the indigenous population?** |
| **4. Would you arrange for a staff member from the same cultural background to care for her if possible?** |

**Do you have any further comments? Thank You./**

---------------------------

## Further general comments

| |
| --- |
| |

**Thank you for your collaboration !**

(For researcher use only: ID Number ___________________)
